# Differential Impact of the COVID-19 Pandemic on Students, Faculty and Staff at a Florida HBCU

**DOI:** 10.1093/geroni/igaa057.3450

**Published:** 2020-12-16

**Authors:** Felicia Wheaton, Matilda Johnson, Thometta Cozart

**Affiliations:** Bethune-Cookman University, Daytona Beach, Florida, United States

## Abstract

The COVID-19 pandemic has caused major disruption to society, including education, the economy, daily life, etc. To understand the impact of the pandemic on both short-term and anticipated long-term mental and physical health, as well as potential period and cohort differences, surveys were emailed to all students, faculty and staff at a Florida HBCU. The survey included the GAD-7 anxiety scale, PHQ-9 scale of depression severity and the UCLA Revised Loneliness Scale (3rd revision), as well as questions about the pandemic’s impact on physical and mental wellbeing in the month of April and long-term physical and mental health. Although loneliness did not differ among groups, students reported the highest levels of moderate/severe depression (46.6%), followed by faculty (21.1%) and staff (6.9%). Students also reported the highest levels of moderate/severe anxiety (48.6%) compared with faculty (29.4%) and staff (12.1%). Students were more likely to say the pandemic moderately or very much impacted their overall physical and mental wellbeing in April. However, faculty were more likely to report that their long-term physical and mental health would be somewhat/greatly affected, followed by students, and then staff. Staff reported the highest levels of optimism about the future, followed by students and faculty. Taken together, these findings indicate substantial differences in the perceived impact of the COVID-19 pandemic.

